# Concept-driven strategies in target-oriented synthesis

**DOI:** 10.3762/bjoc.22.32

**Published:** 2026-03-13

**Authors:** David Yu-Kai Chen, Chao Li, Yefeng Tang

**Affiliations:** 1 Department of Chemistry, Seoul National University, Gwanak-1 Gwanak-ro, Gwanak-gu, Seoul, 08826, South Korea; 2 Tsinghua Institute of Multidisciplinary Biomedical Research, Tsinghua University, 7 Science Park Road ZGC Life Science Park, Beijing,102206, China; 3 National Institute of Biological Sciences, 7 Science Park Road ZGC Life Science Park, Beijing,102206, China; 4 School of Pharmaceutical Sciences, Tsinghua University, 100084 Beijing, China

Humankind’s fascination with organic molecules and their functions holds a special place in the history of science. Indeed, organic compounds have an unparalleled track record in elucidating the mysteries behind a wide range of physical, chemical, and biological phenomena, and advances in chemical synthesis have been instrumental in these explorations. Specifically, target-oriented syntheses of complex organic architectures have witnessed remarkable achievements in recent years, continuously redefining the boundaries of creativity, efficiency, and practicality.

On the other hand, it has been suggested that the field of complex molecular assembly is transitioning (or ought to transition) from an era focused on "feasibility" to one centered around "scalability” [1]. Within this framework, molecular assemblies that integrate elements of "synthetic economy" [2–6] and “sustainability” are highly praised in the synthetic community, driven by methodological breakthroughs, technological advancements, and strategic innovations. However, while efficiency and practicality remain essential design considerations in targeted synthesis, it is worth pondering whether the pursuit of efficiency (along with its associated elements) truly captures the underlying scientific essence of target-oriented synthesis. It is widely acknowledged that the initial allure of target-oriented synthesis often arose from the intellectually and aesthetically captivating strategies involved, with the mastery of synthetic design best exemplified through the perplexing structural relationships between target molecules and their precursors. Moreover, the architectural diversity across a broad spectrum of target molecules both necessitates and inspires the development of strategically distinct approaches tailored to various structural classes, and as a genuine testament to the creativity and vitality of the field. On the other hand, “generalized” design concepts that are applicable across multiple target classes also occupy a prominent role in the synthetic community with resounding successes (Figure 1). Synthetic designs embracing these generalized concepts represent thought processes that complement the paradigms established by the “Corey school” of retrosynthetic analysis [7], particularly in connection with new structural insights and methodological advances. Furthermore, the concurrent implementation of more than one of these concepts may warrant more innovative and effective synthetic designs.

**Figure 1:**
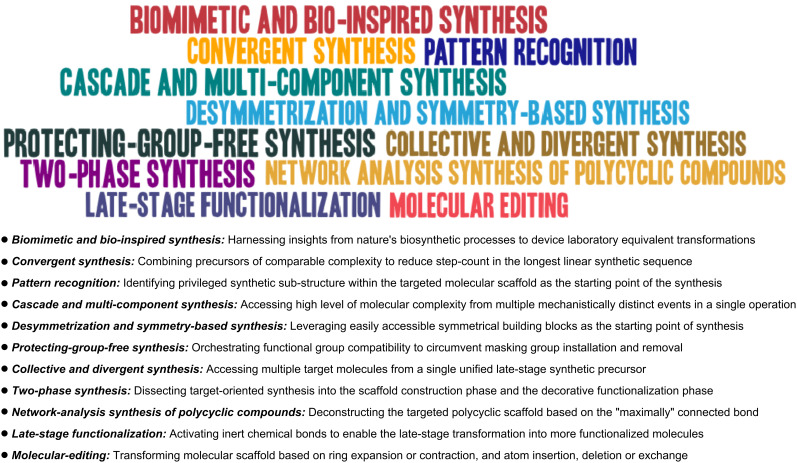
Examples of generalized synthetic concepts featured in target-oriented organic synthesis.

As a related point of discussion, it may be worth comparing synthetic designs based on a “customized” approach with a “generalized” approach (Figure 2). Although a customized approach restricts structural diversity, it has a proven track record and often enables more efficient access to the target molecule. In contrast, a generalized approach can address multiple target classes simultaneously, but typically exhibits reduced efficiency as molecular complexity increases. Integrating elements from both a customized and a generalized approach may represent an “ideal” strategy, although achieving such integration remains a significant challenge.

**Figure 2:**
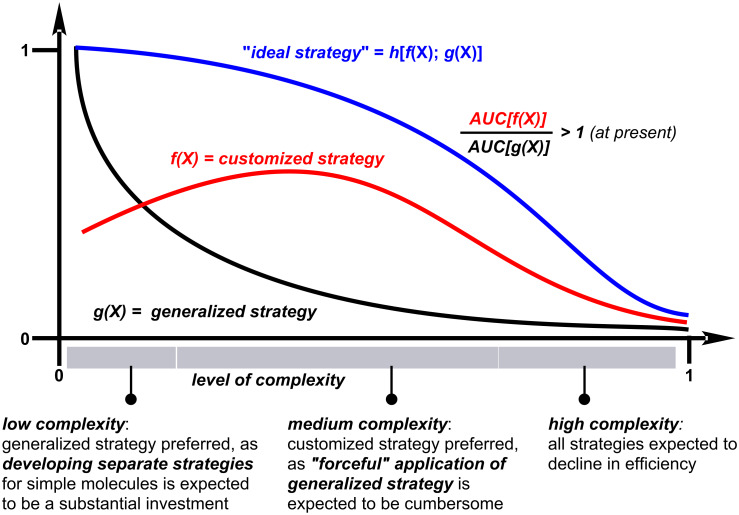
Population-weighted cumulative efficiency (synthetic "planning" and "execution" combined) in “customized”, “generalized” and “ideal” strategies.

In this thematic issue [8], we present a compilation of the latest advances in target-oriented synthesis, contributed by leading experts in the field. These contributions highlight: **(i) strategy-based target-oriented synthesis** [divergent synthesis (review, Leiyang Bai, Xuefeng Jiang and co-worker), convergent synthesis (khayanolide limonoids, Li Zhang, Peirong Rao et al.), stereodivergent synthesis (aspera chaetominines, Pei-Qiang Huang et al.), 1,3-diol desymmetrization (review, Zhifeng Shi, Zhiquiang Ma et al.), 1,3-dicarbonyl reductive desymmetrization (review, Fu-She Han and co-worker), bioinspired synthesis (review, Huilin Li, Xuegong She et al.), biosynthesis (fusicoccane diterpenoids, Yonghui Zhang, Ying Ye, Zheng Xiang et al.), chemoenzymatic synthesis (rhodexin A, Qianghui Zhou et al.)]; **(ii) comparative analysis of target-oriented synthesis** [complanadine A (review, Mingji Dai and co-worker), ryania diterpenoids (review, Jin-Bao Qiao, Yu-Ming Zhao and co-worker), illisimonin A (review, Ming Yang and co-worker)]; and **(iii) methodology towards target-oriented synthesis** [oxidative dearomatization (simonsol C, Hong-Bo Qin et al.), reductive dearomatization (review, Xiangbing Qi and co-worker), enamide cyclization (review, Xiao-Ming Zhang et al.), glycosylation (PI-88, Guothi Xiao et al.), [3 + 2 + 1] cycloaddition (tetrahydrofluorenone, Zhi-Xiang Yu et al.), 1,*n*-enyne cyclization (review, Maosheng Cheng, Lu Yang, Yongxiang Liu et al.), oxidative radical cyclization (prostaglandin D2 metabolite, Jun Huang et al.), reductive cyclization cascade (aglacin B, Jina Xiao, Yu Peng et al.), electrochemical cyclization (review, Bin Li, H. N. C. Wong, Xiao-Shui Peng et al.), photochemical reactions (review, Shao-Min Fu, Bo Liu et al.), carbene insertion (vibralactone, Zhi-Yun Liu, Hong-Dong Hao et al.), interrupted [2 + 2]/retro-Mannich (aspidosperma alkaloids, Zhen Yang, Zhong-Chao Zhang et al.), dipolar cycloaddition (malayamycin A, Sha-Hua Huang, Jian Jin, Ran Hong, et al.), Zr-mediated radical transformations (review, Hugh Nakamura and co-worker)]. The entire editorial team has taken great joy in meticulously overseeing the workflow of each remarkable contribution featured in this thematic issue. Our goal was not only to curate insightful articles but also to foster a sense of community among synthetic enthusiasts at every skill level. We sincerely hope that all readers, whether seasoned experts or newcomers to the field, find as much pleasure and inspiration in these articles as we derived from the preparation and collaboration that brought them to life. We believe that the diverse perspectives and rich discussions encapsulated within these pages will spark interest and deepen understanding in the fascinating world of target-oriented synthesis.

In light of the current scientific landscape, perhaps it is fitting to reflect on the impact of artificial intelligence and innovations in machine-assisted synthetic design as we conclude this Editorial. Nearly four decades ago, Professor Corey noted that "*the field of computer-assisted synthetic analysis is fascinating in its own right and undoubtedly one of the most intriguing challenges in the realm of machine intelligence*." [9]. Since then, we have witnessed remarkable advancements in both the chemical and data sciences. Moreover, the successful translation of these academic breakthroughs into commercial applications has been demonstrated, particularly in the design and prediction of reaction pathways for small organic molecule synthesis [10–12]. The ongoing advances in computational methodology are poised to enhance both accuracy and efficiency, thereby opening new opportunities across various branches of chemical and data sciences. However, while these achievements exemplify the collaborative spirit of scientific inquiry, it is essential to recognize that the primary focus of these research efforts is the identification of “feasible” and “efficient” synthetic pathways. As mentioned in the opening paragraph, as the field of target-oriented synthesis evolves beyond mere feasibility, approaching chemical synthesis solely from a “fast and furious” perspective may not truly capture the essence of this scientific endeavor. It has been said that “*science can give us with what we value, but cannot tell us what we ought to value.*” Perhaps only time will reveal what the future of target-oriented synthesis holds as the community moves forward with great anticipation and excitement.

David Yu-Kai Chen, Chao Li and Yefeng Tang

Seoul and Beijing, March 2026

## Data Availability

Data sharing is not applicable as no new data was generated or analyzed in this study.
